# Transfer of lncRNA UCA1 by hUCMSCs-derived exosomes protects against hypoxia/reoxygenation injury through impairing miR-143-targeted degradation of Bcl-2

**DOI:** 10.18632/aging.202520

**Published:** 2021-02-11

**Authors:** Liwei Diao, Qinghua Zhang

**Affiliations:** 1 Department of Thoracic and Cardiovascular Surgery, University of Chinese Academy of Sciences Shenzhen Hospital, Shenzhen 518000, Guangdong, P.R. China; 2 Department of Cardiovascular Surgery, The Second Affiliated Hospital of Harbin Medical University, Harbin 150086, Heilongjiang, P.R. China

**Keywords:** lncRNA UCA1, exosome, human umbilical cord mesenchymal stem cell, hypoxia/reoxygenation, ischemia-reperfusion

## Abstract

Ischemia results in neuronal damage via alterations in gene transcription and protein expression. Long noncoding RNAs (LncRNAs) are pivotal in the regulation of target protein expression in hypoxia/reoxygenation (H/R). In this study, we observed the function of exosomes-carried lncRNA UCA1 in H/R-induced injury of cardiac microvascular endothelial cells (CMECs). In H/R cell model, CMECs were co-cultured with human umbilical cord mesenchymal stem cell-derived exosomes (hUCMSC-ex). The loss-of-function experiments were conducted to assess the effect of lncRNA UCA1 on H/R injury by assessing the biological behaviors of CMECs. The relationship among lncRNA UCA1, miR-143 and Bcl-2 were verified. An ischemia-reperfusion (I/R) rat model was established. Then hUCMSC-ex was injected into I/R rats to identify its effects on apoptosis and autophagy. Functional rescue experiments were performed to verify the sponge system. *In vitro* and *in vivo* experiments showed that hUCMSC-ex protected I/R rats and H/R CMECs against injury. Silencing UCA1 in hUCMSC-ex or miR-143 overexpression aggravated H/R injury in CMECs. LncRNA UCA1 competitively bound to miR-143 to upregulate Bcl-2. And hUCMSCs-ex/si-UCA1+inhi-miR-143 treatment protected CMECs against H/R injury and inhibited hyperautophagy. Together, hUCMSC-ex-derived lncRNA UCA1 alleviates H/R injury through the miR-143/Bcl-2/Beclin-1 axis. Hence, this study highlights a stem cell-based approach against I/R injury.

## INTRODUCTION

Endothelial hypoxia/reoxygenation (H/R) is a main consequence of ischemia-reperfusion (I/R) injury that triggers oxidative stress with increased release of superoxide and reduced generation of nitric oxide, resulting in endothelial dysfunction [1]. H/R causes tissue injury in diverse organs and induces cell apoptosis [2]. Multiple pathways are implicated in the pathogenesis of I/R injury, including ion channels, inflammatory responses, oxidative stress and endothelial dysfunction [3]. Prevention of acute I/R injury exerts a potential to improve clinical prognosis for patients suffering from acute myocardial infarction [4]. Extracellular vesicles including exosomes, microvesicles and apoptotic bodies are emerged as mediators of cell-cell communication in different pathophysiological settings, unraveling new therapeutic avenues for the treatment of cardiovascular diseases [5]. Exosomes are vesicles in nanometer size that involve in the transfer of signals throughout the body and have cardioprotective effects on cardiac I/R injury in all tested models [6]. One important finding is that exosomes released from mesenchymal stromal cells protect against mouse lung I/R injury *via* transfer of anti-apoptotic microRNA (miR), miR-21-5p [7]. However, the specific mechanisms underlying the cellular function of exosomes in I/R or H/R injury are urgent to be further discovered.

Mesenchymal stem cells (MSCs) derived from human umbilical cord (hucMSCs) are able to regulate tissue repair and regeneration [8]. MSCs could secrete several kinds of exosomes to exert their effects [9]. Exosomes contain a broad range of nucleic acids such as mRNA and long non-coding RNAs (lncRNAs) [10]. LncRNAs are transcripts (more than 200 base pairs) without protein-coding potential, and are functional in cellular function and tissue homeostasis in cardiovascular diseases [11]. LncRNAs function as modulators of endothelial cell proliferation and vascular smooth muscle cell (VSMC) phenotypes [12]. For instance, lncRNA H19 is identified as an inducer of H/R injury by enhancing autophagy in hepatoma carcinoma cells [13]. Additionally, miRs are commonly known to induce degradation of target genes *via* binding to their mRNAs [14]. Interestingly, lncRNA UCA1, enriched in the heart, could reduce H/R-induced apoptosis of cardiomyocytes through inhibition of miR-143 and mediating MDM2/p53 signaling pathway [15].

Although the role of the lncRNA UCA1/miR-143 axis has been disclosed, transfer of lncRNA UCA1 *via* exosomes into recipient cells and functions of exosomal lncRNA UCA1 in I/R or H/R injury remain unknown. We intended to explore whether human umbilical cord mesenchymal stem cell-derived exosomes (hUCMSC-ex) could transfer lncRNA UCA1 into cardiac microvascular endothelial cells (CMECs) to regulate miR-143 and to exert protective functions in I/R animal and H/R cell models.

## RESULTS

### Successfully isolation of hUCMSCs and hUCMSC-ex

**Figure 1:**
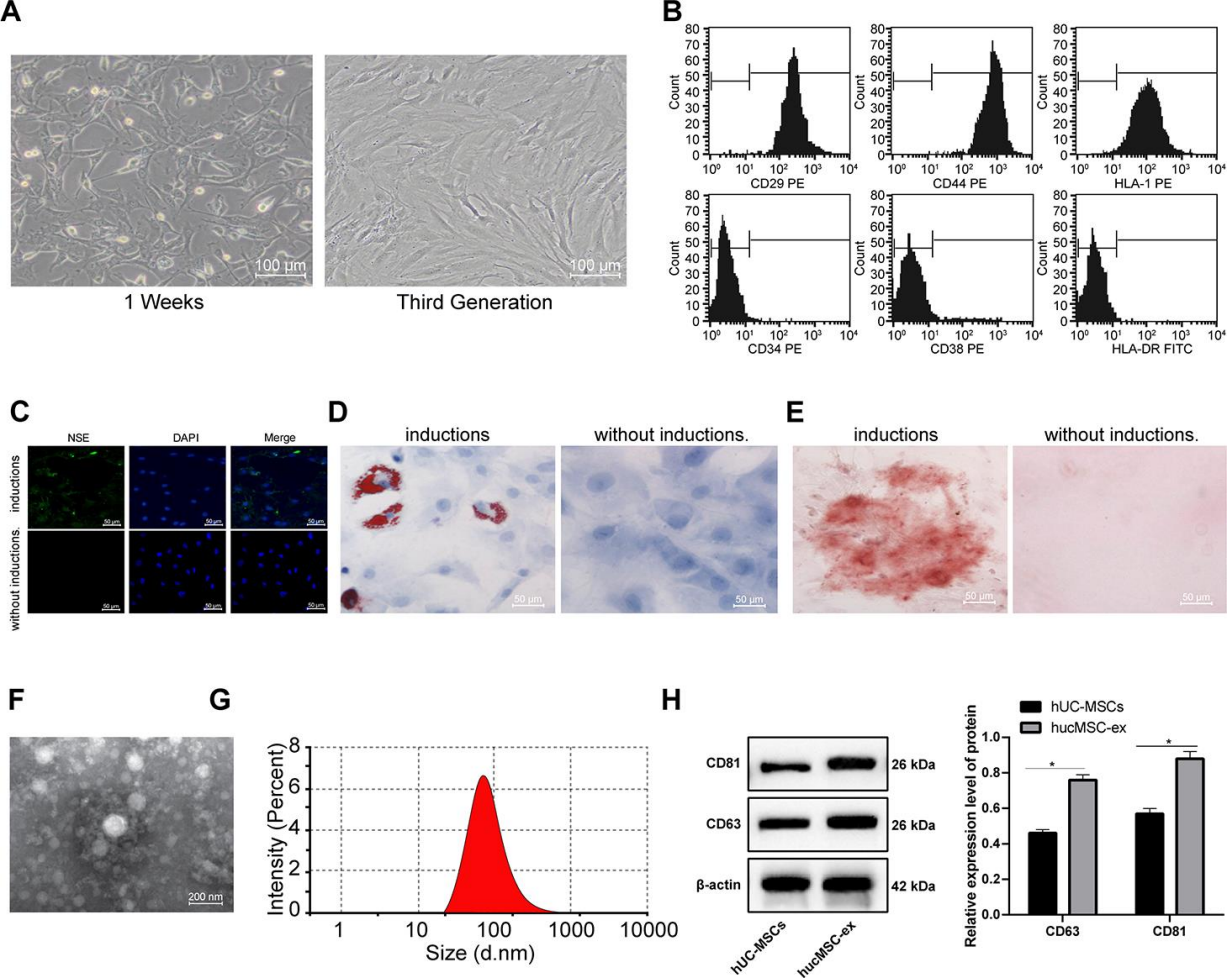
**The hUCMSCs and their derived hUCMSC-ex are successfully isolated.** (**A**) Identification of hUCMSCs after 1 week of culture and at passage 3. (**B**) Expression of mesenchymal cell surface markers (CD29, CD44, HLA-I, CD34, CD38 and HLA-DR) detected by flow cytometry. (**C**) Immunofluorescence assay detected the neurogenic induction of hUCMSCs. (**D**) Oil red O staining measured the adipogenic induction of hUCMSCs. (**E**) Alizarin red staining measured the osteogenic induction of hUCMSCs. (**F**) TEM observation of morphology of exosomes. (**G**) Particle size of exosomes calculated using ZETASIZER Nano series-Nano-ZS. (**H**) Protein expression of CD63 and CD81 in isolated hUCMSC-ex measured by Western blot assay. All the experiments were repeated 3 times. Data in panel (**H**) were analyzed by two-way ANOVA and the pairwise comparisons after ANOVA were performed with Tukey's multiple comparisons test. *p < 0.05.

When hUCMSCs were isolated and cultured for 1 week, the cells at the edge of the tissue blocks were spindle-shaped. After 2 weeks, a large number of colonies were observed, in which the cells were spindle-shaped and three-dimensional spiral-shaped. When the cell confluence reached 80%-90%, cells were passaged. The morphology of cells at passage 3 was uniform and full (Figure 1A). Flow cytometry detected the mesenchymal cell surface markers, and displayed that cells were positive for CD29, CD44 and HLA-I, and negative for CD34, CD38 and HLA-DR (Figure 1B).

hUCMSCs were induced for neurogenic, adipogenic and osteogenic differentiation. After induction, hUCMSCs were differentiated into a great number of neuron-like cells, and the lipid droplets increased and gradually merged into lipid vesicles, and a large number of reddish calcium nodules were seen (Figure 1C–1E).

The hUCMSCs were collected and exosomes were isolated from using ultra-centrifugation. TEM observation showed that the exosomes were round-shaped or oval-shaped with a bilayer membrane structure (Figure 1F). Nanosight nanoparticle tracking analyzer measured that the diameter of exosomes was 30-150 nm (Figure 1G). Western blot assay found that highly expressed CD61 and CD81 in purified exosomes (Figure 1H), suggesting that high-purity exosomes were extracted from hUCMSCs.

### Protective role of hUCMSC-ex in CMECs against H/R injury

**Figure 2:**
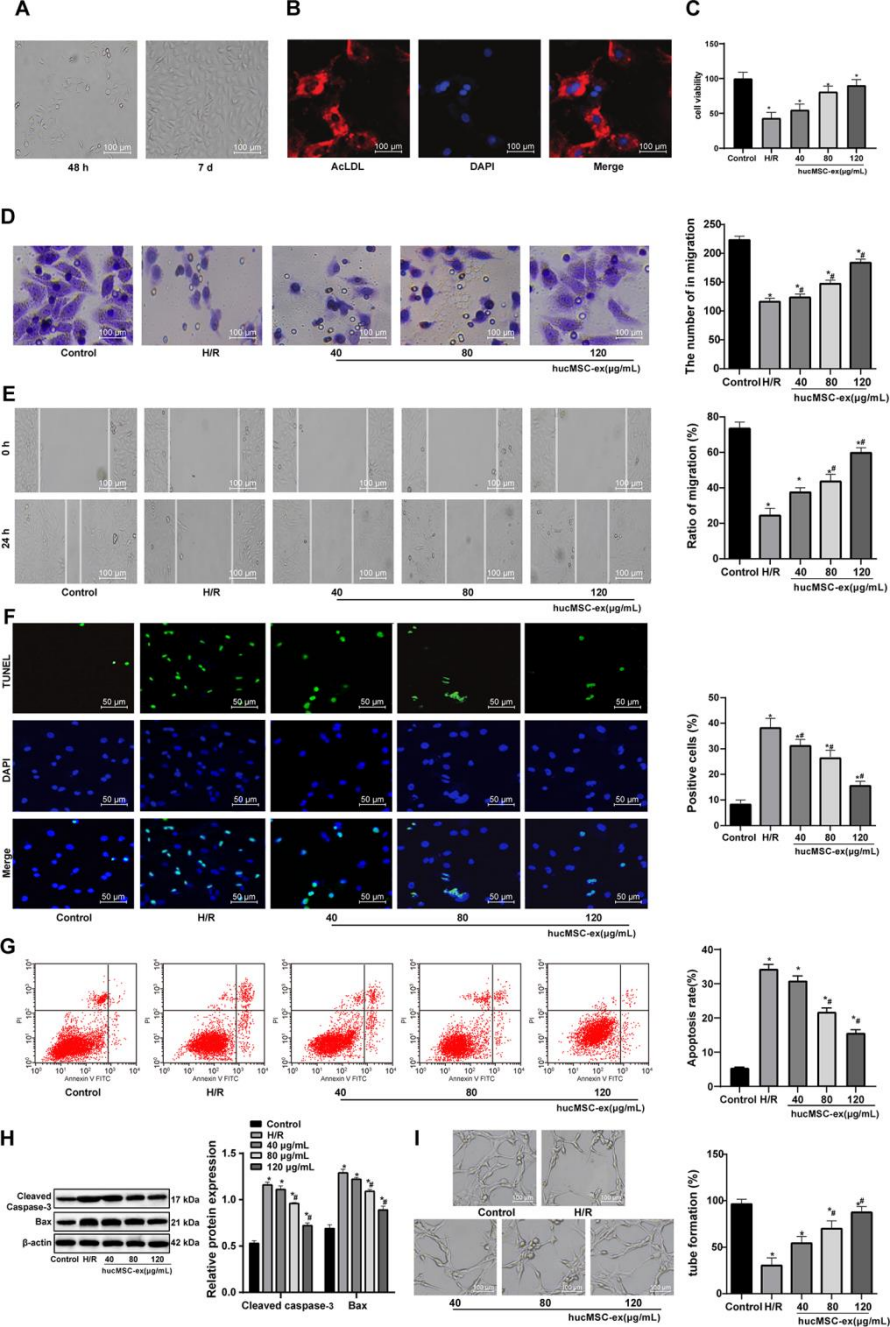
**The hUCMSC-ex facilitates growth and restrains apoptosis of H/R-damaged CMECs in a concentration-dependent manner.** (**A**) CMECs observed under microscope after 48 h (left) and at the 5-7^th^ d (right) of cell culture. (**B**) Dil-ac-LDL-positive CMECs (red) and DAPI-positive (blue) under fluorescence microscope after 7 d of cell culture. (**C**) Viability of CMECs detected by CCK-8 after co-culture with hUCMSC-ex. (**D**) Invasion of CMECs assessed by Transwell assay after co-culture with hUCMSC-ex. (**E**) Migration of CMECs evaluated by scratch test after co-culture with hUCMSC-ex. (**F**) The number of TUNEL-positive CMECs after co-culture with hUCMSC-ex. (**G**) Apoptotic rate of CMECs assessed by flow cytometry after co-culture with hUCMSC-ex. (**H**) Expression of apoptosis-related proteins Cleaved caspase3 and Bax in CMECs after co-culture with hUCMSC-ex measured by Western blot assay. (**I**) Tube formation in CMECs after co-culture with hUCMSC-ex. *p < 0.05, *vs*. the control group; #*p* < 0.05 *vs*. the H/R group. All experiments were repeated 3 times. Data in panels (**C**–**G**, **I**) were analyzed by one-way ANOVA, and data in panel (**H**) were analyzed by two-way ANOVA, and the pairwise comparisons after ANOVA were performed with Tukey’s multiple comparisons test.

According to a previous literature, hUCMSC-ex promotes endothelial cell growth, migration and tube formation [16]. Therefore, we speculate that hUCMSC-ex may also intervene the biological behaviors of CMECs injured by H/R. First, we cultured primary CMECs for 48 h, and saw that CMECs were small and short spindle-shaped. As the culture days increased, the number of adherent cells gradually increased, and tended to stretch and became larger. At the 5-7^th^ d, the adherent cells were short, fusiform- or polygon-shaped, uniform in size, and the monolayers were arranged in a paving stone-like structure (Figure 2A). At the 7^th^ d, cells were positive for Dil-ac-LDL (red) and DAPI (blue) under fluorescence microscope, which could be identified as CMECs (Figure 2B), suggesting that the isolated CMECs were not contaminated by cardiomyocytes or fibroblasts, and that CMECs were in high purity and can be used in the next experiments.

After the establishment of H/R model, the viability, invasion and migration abilities of CMECs were found to be elevated with the increase of hUCMSC-ex concentrations, suggestive of a regulation in a concentration-dependent manner. When hUCMSC-ex concentration was 80 μg/mL, CMEC viability was maximum (Figure 2C–2E). In addition, with the increase of hUCMSC-ex concentrations, TUNEL-positive CMECs, apoptotic rate and levels of apoptosis-related proteins Cleaved caspase3 and Bax decreased remarkably, while the number of tube formation increased significantly in a concentration-dependent manner (all *p* < 0.05) (Figure 2F–2I). Together, those data suggested that hUCMSC-ex at 80 μg/mL was best protective for CMECs from H.R injury. Therefore, hUCMSC-ex at 80 μg/mL was selected for the subsequent experiments.

### Protective role of hUCMSC-ex in rats against I/R injury

**Figure 3:**
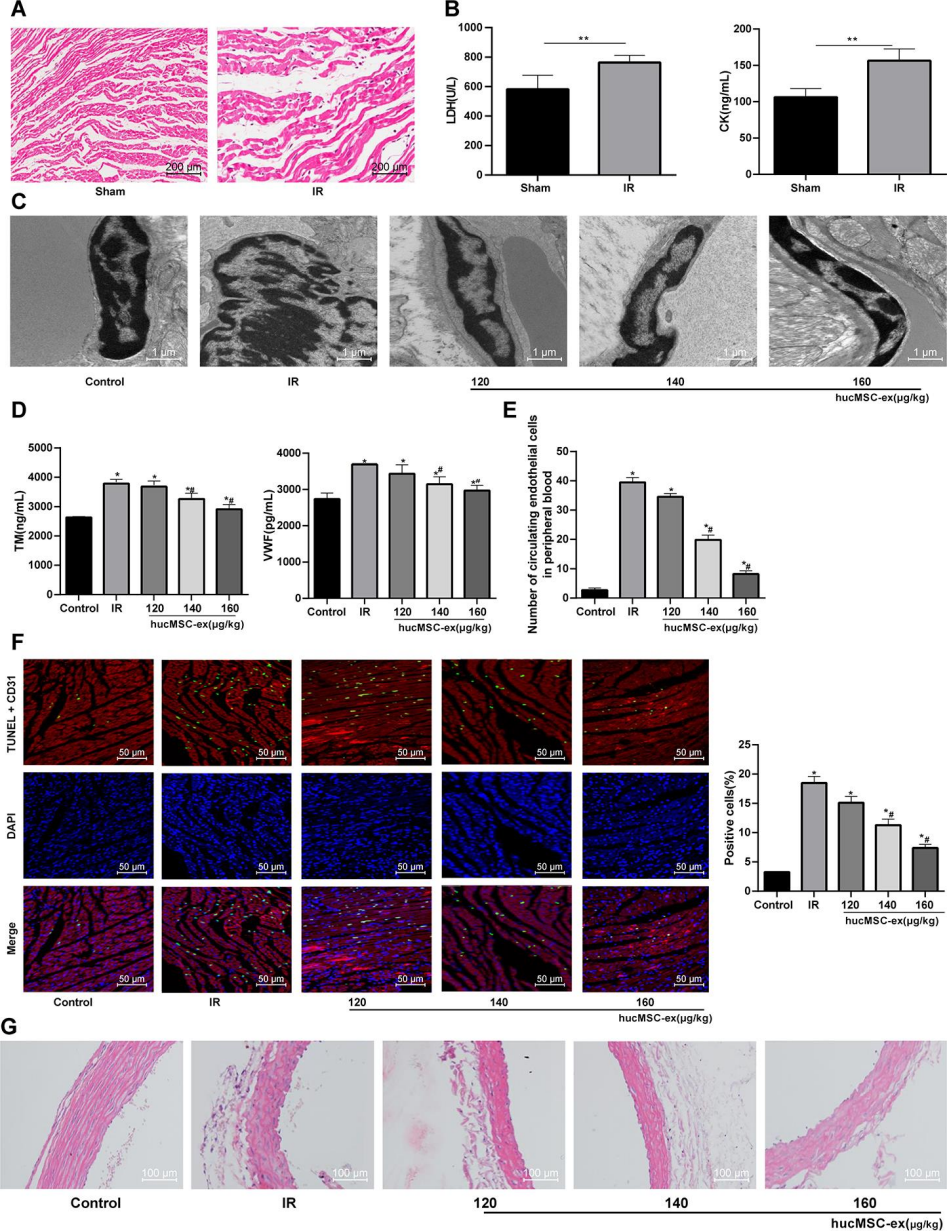
**The hUCMSC-ex protects rats against the I/R injury.** (**A**) HE staining for the detection of myocardial pathological morphology in rats injected with different concentrations of hUCMSC-ex (n = 3). (**B**) Levels of cardiac function indexes LDH and CK in I/R rats injected with different concentrations of hUCMSC-ex measured by ELISA kits (n = 8). (**C**) Ultrastructure of CMECs in I/R rats injected with different concentrations of hUCMSC-ex observed under transmission electron microscope (n = 3). (**D**) Serum TM and vWF levels in I/R rats injected with different concentrations of hUCMSC-ex measured by ELISA kits (n = 8). (**E**) The number of CECs in I/R rats injected with different concentrations of hUCMSC-ex measured by flow cytometry (n = 8). (**F**) CD31 + TUNEL-positive CMECs in I/R rats injected with different concentrations of hUCMSC-ex (n = 3). CMECs were red labeled by CD31, TUNEL-positive cells were green, and DAPI was blue. (**G**) HE staining of arterial intima structure in I/R rats injected with different concentrations of hUCMSC-ex (n = 3). *p < 0.05, *vs*. the control group; #*p* < 0.05, *vs*. the H/R group. All experiments were repeated 3 times. Data in panel (**B**) were analyzed with independent t test, and data in panels (**E, F** and **G**) were analyzed by one-way ANOVA and the pairwise comparisons after ANOVA were performed with Tukey’s multiple comparisons test.

To explore the effect of hUCMSC-ex *in vivo*, we carried out *in vivo* experiments and constructed the rat model of I/R. Obvious broken myocardial fibers and swelling cells were observed in rats following I/R (Figure 3A); and ELISA kits showed that cardiac function indexes LDH and CK were significantly increased after I/R (both *p* < 0.05) (Figure 3B), suggesting successful development of I/R rat model.

I/R rat models were injected with different concentrations of hUCMSC-ex. TEM showed enlarged nuclei of endothelial cells from I/R rats, with finger-like membrane protruding into the lumen. With the concentrations of hUCMSC-ex increased, the ultrastructure of rat CMECs was gradually improved (Figure 3C). Additionally, vWF and TM are two markers reflecting the degree of vascular endothelial injury. To determine the injury of myocardial endothelium in each group, vWF and TM levels were measured. TM and vWF levels in sera were significantly increased in I/R rats, both of which were gradually lowered by the increased concentrations of hUCMSC-ex (all *p* < 0.05) (Figure 3D). Flow cytometry indicated that the number of CECs was significantly increased in I/R rats, but decreased gradually and vascular endothelial injury was alleviated with the increase concentrations of hUCMSC-ex (both *p* < 0.05) (Figure 3E). Then, in order to evaluate the apoptosis of CMECs in each group, we carried out immunofluorescence double staining, using CD31 (red) to label CMECs, TUNEL (green) to label apoptotic cells. Compared with the controls, the CD31 + TUNEL positive signal of CMECs in I/R rats was increased significantly, which was gradually decreased with the addition of hUCMSC-ex (Figure 3F). HE staining showed disordered arterial intima structure, endothelium convex and defect in the I/R rats, and the structure, thickness and intima integrity of the thoracic aorta were improved to some degrees with the addition of hUCMSC-ex (Figure 3G).

### Inhibitory effects of hUCMSC-ex on CMECs autophagy in H/R

**Figure 4:**
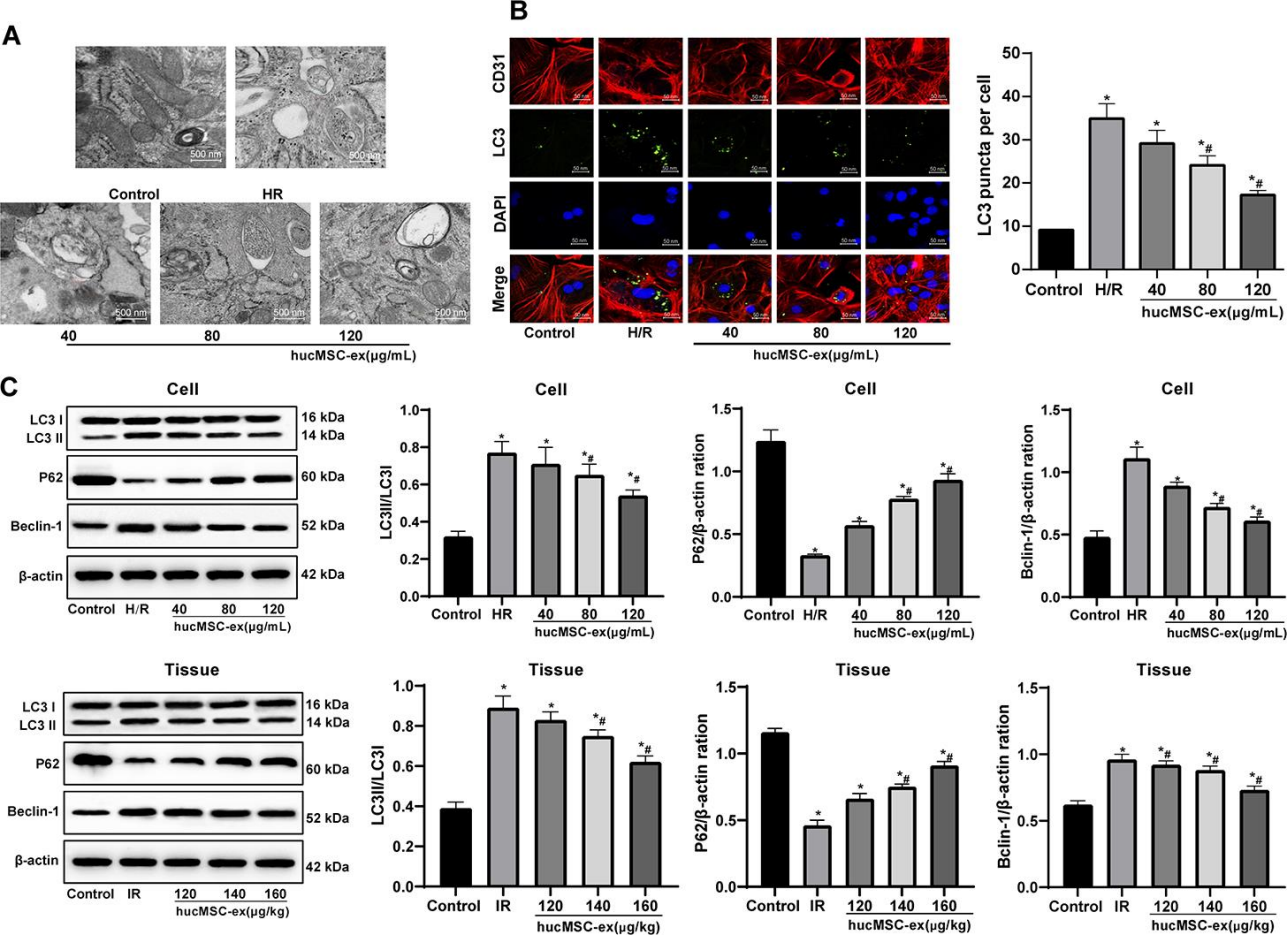
**The hUCMSC-ex protects CMECs against autophagy following H/R injury.** (**A**) Transmission electron microscope observation of autophagosomes; (**B**) Fluorescence localization of LC3 in CMECs under exposure to H/R injury. LC3 was labeled as green fluorescence, nucleus were labeled by DAPI and CMEC marker CD31 was labeled as red fluorescence. (**C**) LC3-II/LC3-I, Beclin-1 and p62 expression in CMECs under exposure to H/R injury measured by Western blot assay. *p < 0.05, *vs*. the control group; #*p* < 0.05, *vs*. the H/R or I/R group. All experiments were repeated 3 times. Data in panels (**B** and **C**) were analyzed with one-way ANOVA and Tukey’s multiple comparisons test.

Autophagy is protective for myocardium during the ischemic process, but aggravates cell death in the reperfusion process [17]. To investigate the effect of hUCMSC-ex on autophagy of H/R injured CMEC, we observed the formation of autophagosomes by TEM *in vitro*. In the H/R CMEC model, autophagosomes gradually decreased by the increase concentrations of hUCMSC-ex, corresponding to reductions in LC3 fluorescence expression and LC3-II/LC3-I and Beclin-1 protein levels, as well as an increase in p62 protein level (all *p* < 0.05) (Figure 4A–4C). In conclusion, hUCMSC-ex suppresses autophagy in CMECs under exposure to H/R.

### Inhibition lncRNA UCA1 in hUCMSC-ex aggravates H/R-induced CMEC injury

**Figure 5:**
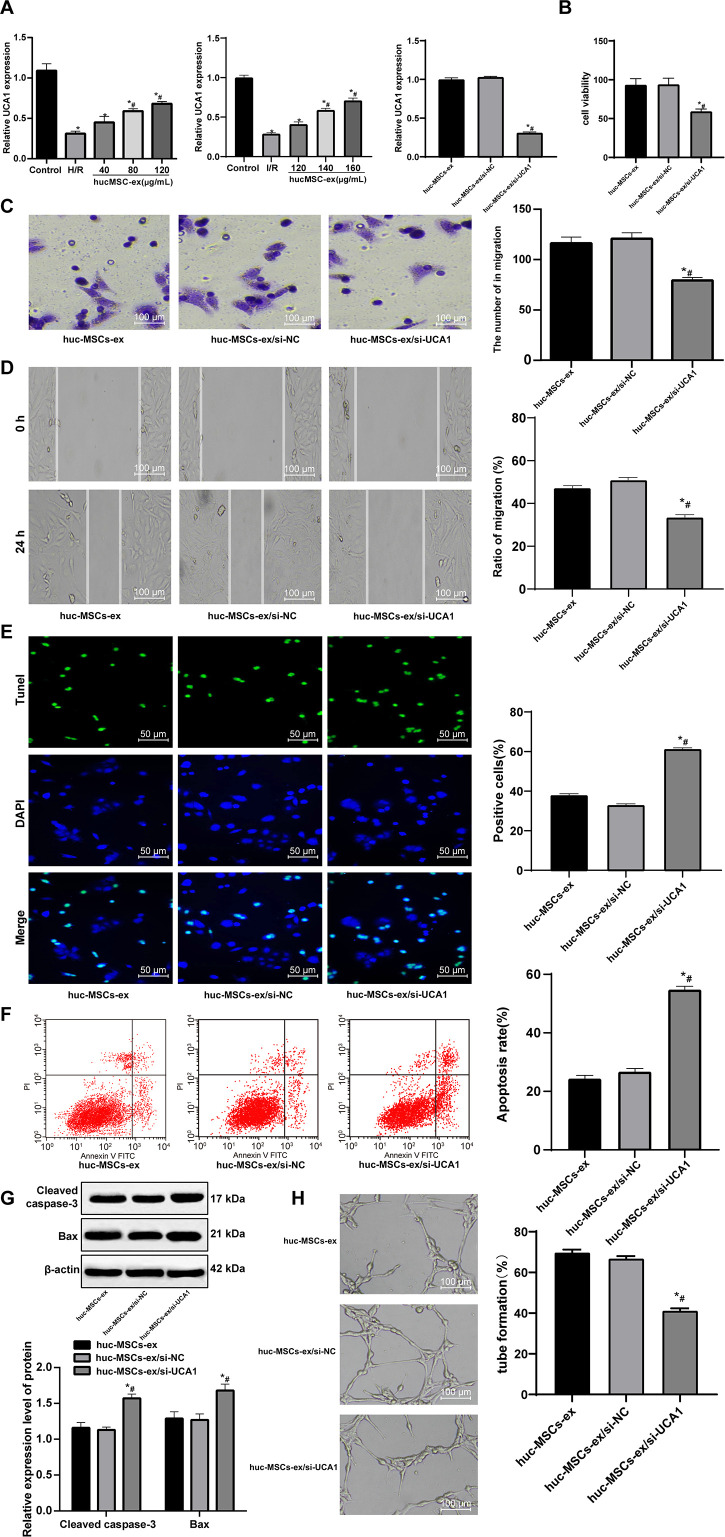
**LncRNA UCA1 silencing in hUCMSC-ex facilitates H/R injury in CMECs.** (**A**) LncRNA UCA1 expression in CMECs determined by RT-qPCR; (**B**) Viability of CMECs detected by CCK-8. (**C**) Invasion of CMECs assessed by Transwell assay. (**D**) Migration of CMECs evaluated by scratch test after co-culture with hUCMSC-ex. (**E**) The number of TUNEL-positive CMECs. (**F**) Apoptotic rate of CMECs assessed by flow cytometry. (**G**) Expression of apoptosis-related proteins Cleaved caspase3 and Bax in CMECs measured by Western blot assay. (**H**) Tube formation in CMECs. *p < 0.05, *vs*. the control or hUCMSCs-ex group; #*p* < 0.05, *vs*. the H/R group. All experiments were repeated 3 times. Data in panel (**G**) were analyzed with two-way ANOVA, and data in other panels were analyzed with one-way ANOVA, followed by Tukey’s multiple comparisons test.

Recently, exosomes are emerged as vehicles to deliver lncRNAs to recipient cells [18]. LncRNA UCA1 is protective against myocardial cell injury induced by anoxia and glucose deficiency [19]. In light of these, we speculated that hUCMSC-ex might play a protective role in H/R injury of CMECs by delivering lncRNA UCA1. To verify this idea, we used RT-qPCR to detect the expression of lncRNA UCA1 in cardiomyocytes and myocardial tissues. Compared with the controls, after H/R injury, the expression of lncRNA UCA1 in cardiomyocytes and myocardial tissues decreased significantly. With the increasing concentrations of hUCMSC-ex, the expression of lncRNA UCA1 increased gradually. It can be seen that the exosomes released lncRNA UCA1 into cells. To verify that exosomes are protective by releasing lncRNA UCA1, we successfully interfered with the expression of UCA1 (Figure 5A) in hUCMSCs, and extracted the exosomes for *in vitro* experiments. CCK-8, Transwell, scratch, TUNEL, flow cytometry, Western blot and tubule formation assays were used to evaluate the biological episodes of cardiomyocytes. The results showed that the protective effect of hUCMSC-ex/si-UCA1 on CMEC H/R injury was weakened (all *p* < 0.05) (Figure 5B–5H).

### LncRNA UCA1 competitively binds to miR-143 and Bcl-2 is a direct target of miR-143

**Figure 6:**
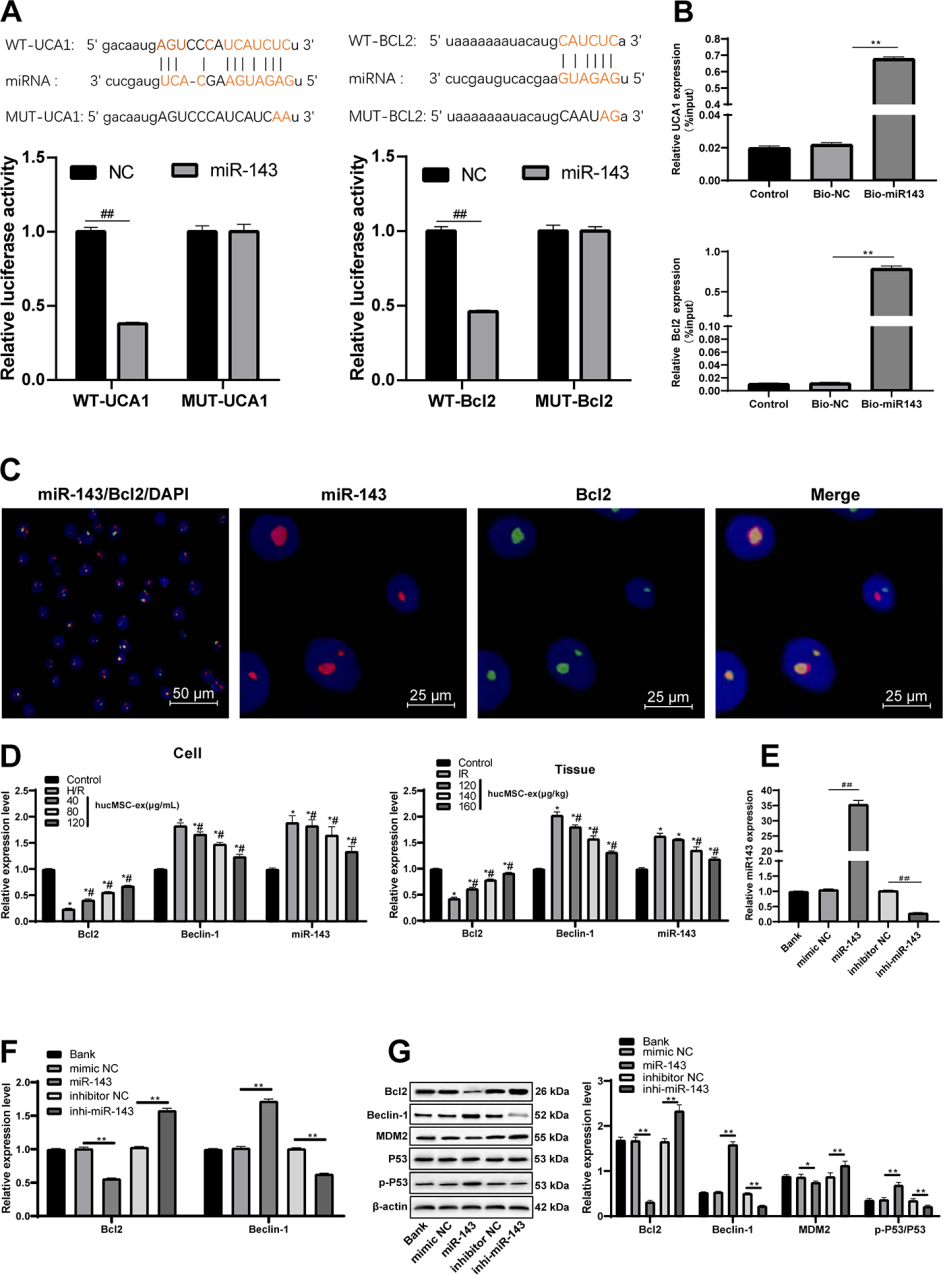
**LncRNA UCA1 could competitively bind to miR-143 to upregulate Bcl-2.** (**A**) Dual luciferase reporter gene assay verified the binding relationship between lncRNA UCA1 and miR-143, and Bcl-2 and miR-143. (**B**) RNA pull-down verified the binding relationship between lncRNA UCA1 and miR-143, and Bcl-2 and miR-143. (**C**) Two-color fluorescence in-situ hybridization verified the binding relationship between Bcl-2 and miR-143. The probe of miR-143 was labeled with red fluorescence, the probe of BCL2 was labeled with green fluorescence, and the yellow fluorescence was the overlapping of miR-143 and Bcl2, indicating that miR-143 and BCL2 are directly related. (**D**) Expression of lncRNA UCA1, miR-143, Bcl-2 and Beclin-1 in cardiomyocytes and myocardial tissue. (**E**, **F**) After miR-143 mimic or inhibitor transfection, expression of miR-143, Bcl-2 and Beclin-1 was determined by RT-qPCR; (**G**) Bcl-2 and Beclin-1 protein levels and MDM2/p53 pathway related protein levels were measured Western blot assay. *p < 0.05, *vs*. the control group; #*p* < 0.05, *vs*. the H/R group. All experiments were repeated 3 times. Data in panels (**B** and **E**) were analyzed with one-way ANOVA, and data in panels (**A**, **D**, **F** and **G**) were analyzed with two-way ANOVA, followed by Tukey’s multiple comparisons test.

As previously reported, lncRNA UCA1 controls mRNA degradation by RNA-RNA competitive interactions [20]. Atorvastatin inhibits miR-143 expression to exert protection against oxidative stress in cardiomyocytes [21]. Therefore, we speculated that lncRNA UCA1 might also protect against H/R injury in cardiomyocytes by binding to miR-143. The binding relationship between lncRNA UCA1 and miR-143 was predicted through the biological website (https://web.archive.org/web/20110222111721/http://starbase.sysu.edu.cn/), and further verified by dual luciferase reporter gene assay and RNA pull-down experiment (Figure 6B). However, the downstream mechanism of miR-143 was unknown. Increasing the expression of Bcl-2 and decreasing the expression of Beclin1 protect against I/R injury [22]. Therefore, we speculate that Bcl-2 and Beclin1 may interact with miR-143. It was predicted a binding relationship between miR-143 and Bcl-2 by the biological website. Then, the binding relationship was further verified by dual luciferase reporter gene assay, RNA pull-down and two-color fluorescence in-situ hybridization (Figure 6A–6C). The expression of lncRNA UCA1 and Bcl-2 was upregulated with the increase concentrations of hUCMSC-ex, while miR-143 and Beclin-1 expression was downregulated (all *p* < 0.05) (Figure 6D). After transfection with miR-143 mimic, the expression of miR-143 and Beclin-1 was upregulated, and Bcl-2 was downregulated, opposite to the changes caused by miR-143 inhibitor transfection (Figure 6E). The results suggested that hUCMSC-ex transmits lncRNA UCA1 to CMECs to competitively bind miR-143 to reduce the targeting inhibition of miR-143 on Bcl2. In addition, it is reported that lncRNA UCA1 can protect cardiomyocytes from apoptosis induced by H/R by inhibiting miR-143 and regulating downstream MDM2/p53/BCL2 signaling pathway [14]. Therefore, Western blot was used to detect the levels of MDM2/p53 pathway related proteins. After overexpression of miR-143, the level of p-p53/p53 was upregulated, and the level of MDM2 was downregulated, while the low expression of miR-143 showed the opposite trends (all *p* < 0.05) (Figure 6F–6G). However, after intervention of miR-143, BCL2 showed more significant changes compared with the levels of MDM2/p53 pathway related proteins. In conclusion, UCA1 transferred by hUCMSC-ex as sponge adsorbed miR-143, and then regulated the expression of BCL2 to protect CMECs from H/R injury.

### Upregulation of miR-143 aggravates H/R-induced CMEC injury

**Figure 7:**
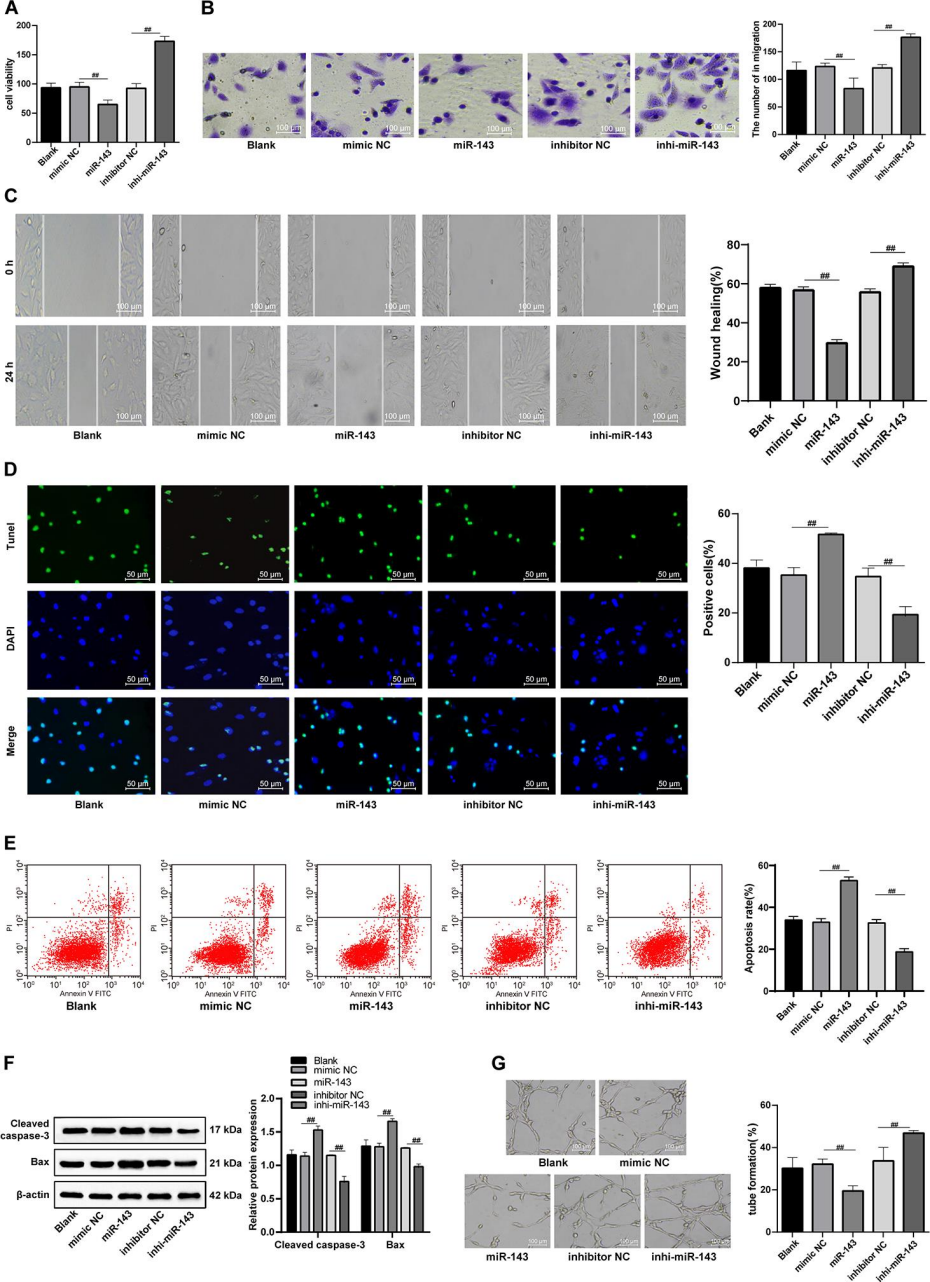
**The hUCMSC-ex protects CMECs against H/R injury.** (**A**) Viability of CMECs transfected with miR-143 mimic or inhibitor detected by CCK-8. (**B**) Invasion of CMECs transfected with miR-143 mimic or inhibitor assessed by Transwell assay. (**C**) Migration of CMECs transfected with miR-143 mimic or inhibitor evaluated by scratch test. (**D**) The number of TUNEL-positive CMECs transfected with miR-143 mimic or inhibitor. (**E**) Apoptotic rate of CMECs transfected with miR-143 mimic or inhibitor assessed by flow cytometry. (**F**) Expression of apoptosis-related proteins Cleaved caspase3 and Bax in CMECs transfected with miR-143 mimic or inhibitor measured by Western blot assay. (**G**). Tube formation in CMECs transfected with miR-143 mimic or inhibitor.*p < 0.05, *vs*. the control group; #*p* < 0.05, *vs*. the H/R group. All experiments were repeated 3 times. Data in panel (**F**) were analyzed with two-way ANOVA, and data in other panels were analyzed with one-way ANOVA, followed by Tukey’s multiple comparisons test.

After overexpression of miR-143, the viability, invasion and migration of H/R-injured CMECs were dramatically decreased (all *p* < 0.05), all of which were enhanced by inhibition of miR-143 (Figure 7A–7C). In addition, TUNEL-positive CMECs and apoptosis rate, levels of Cleaved caspase3 and Bax were increased remarkably, and the lumen formation was inhibited by overexpression of miR-143 (all *p* < 0.05) (Figure 7D–7G), opposite to the changes in those caused by inhibition of miR-143. In summary, miR-143 overexpression deteriorates H/R injury in CMECs.

### Exosomal lncRNA UCA1 inhibits hyperautophagy and alleviates H/R-induced CMEC injury by impairing miR-143-targeted degradation of Bcl-2

**Figure 8:**
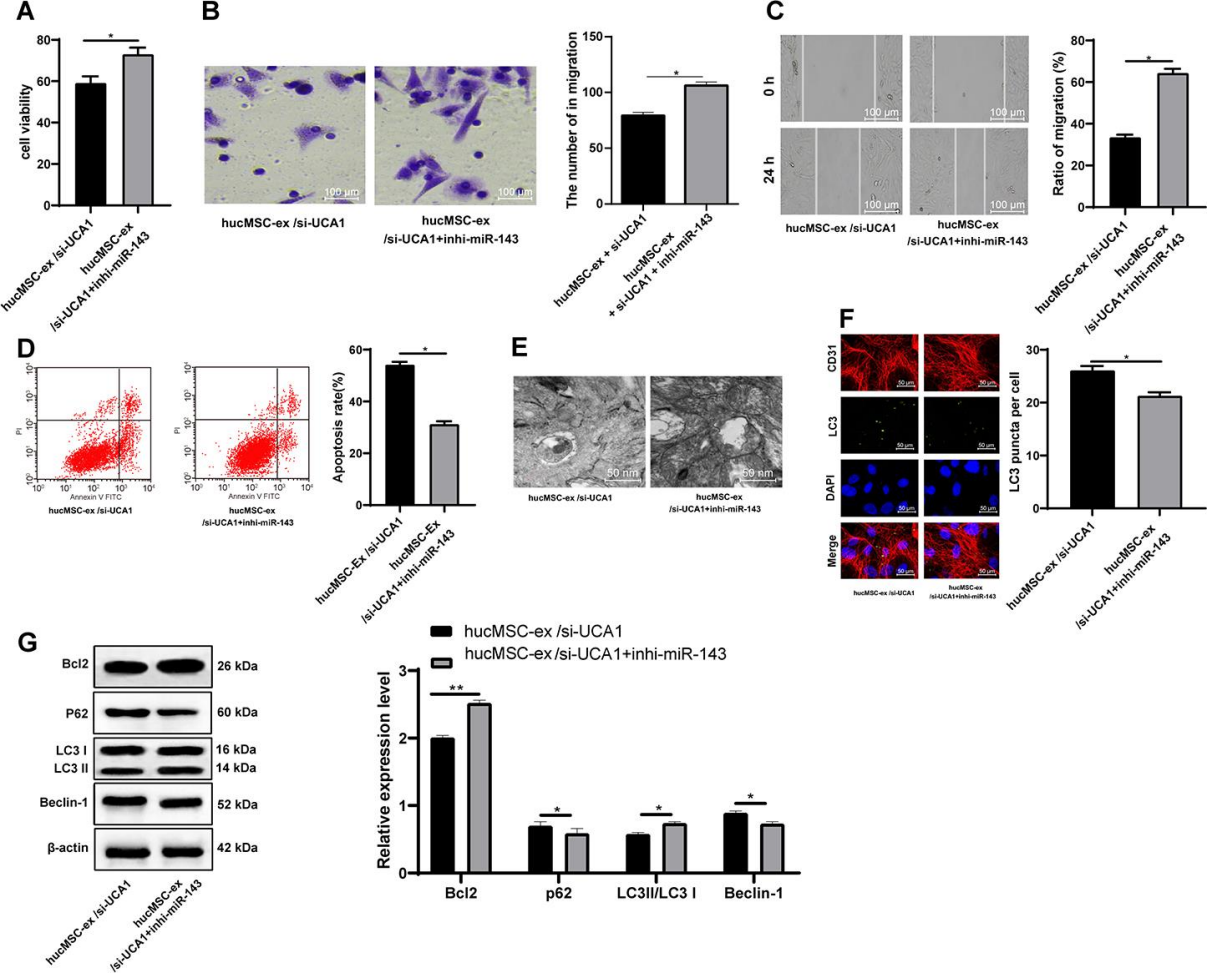
**LncRNA UCA1 in hUCMSC-ex protects CMECs against autophagy following H/R injury through impairing miR-143-targeted inhibition of Bcl-2.** (**A**) Viability of CMECs detected by CCK-8. (**B**) Invasion of CMECs assessed by Transwell assay. (**C**) Migration of CMECs evaluated by scratch test. (**E**) Transmission electron microscopic observation of autophagosomes; (**F**) Fluorescence localization of LC3 in CMECs under exposure to H/R injury. LC3 was labeled as green fluorescence, nucleus were labeled by DAPI and CMEC marker CD31 was labeled as red fluorescence. (**G**) LC3-II/LC3-I and Beclin-1 protein levels in CMECs under exposure to H/R injury measured by Western blot assay. *p < 0.05, *vs*. the control group; # *p* < 0.05, *vs*. the H/R group. All experiments were repeated 3 times. Data in panels (**A**–**G**) were analyzed with independent t test.

To further confirm the role of lncRNA UCA1 through the adsorption of miR-143, we carried out a functional rescue experiment, by setting up the cell group of exosomes after interfering with lncRNA UCA1 (hUCMSCs-ex/si-UCA1), and the combined group of hUCMSCs-ex/si-UCA1 and low expression miR-143 (hUCMSCs-ex/si-UCA1 + inhi-miR-143). Compared with the hUCMSCs-ex/si-UCA1 treatment, hUCMSCs- ex/si-UCA1 + inhi-miR-143 treatment elevated the viability, invasion and migration of H/R-injured CMECs, reduced apoptotic rate and autophagosomes, LC3 fluorescence, LC3-II/LC3-I and Beclin-1 protein levels and increased p62 protein level (all *p* < 0.05) (Figure 8A–8G).

## DISCUSSION

This study mainly paid attention to the potential of exosomal lncRNA UCA1 in H/R injury of CMECs. All data together confirmed hUCMSC-ex as a carrier that could transfer lncRNA UCA1 into H/R-injured CMECs, and exert its protective effect against H/R injury through the miR-143/Bcl-2/Beclin-1 axis.

The hUCMSCs are widely applied to treat several diseases. Intravenous transplantation of hUCMSCs could promote the behavioral recovery of hypoxic-ischemic rats in early hypoxic-ischemic encephalopathy [23]. An angiogenic effect of hUCMSCs has been reported in stroked brain [24]. First of all, our study evidenced that hUCMSC-ex enhanced the proliferation, invasion and migration, and inhibited apoptosis and autophagy of H/R-damaged CMECs in a concentration-dependent manner. Inhibition of apoptosis and autophagy by inducing the expression of Bcl-2 and inhibiting the levels of Bax and beclin-1 is protective against H/R injury in the H9c2 myocytes [25]. The protective effects were also verified in an *in vivo* I/R model, presented with a reduction in the apoptosis of CECs as well as TM and vWF serum levels. Administration of hUCMSC-ex protects cardiomyocytes against apoptosis and promotes tube formation and migration of umbilical vein endothelial cells [26], which are similar to the protective effect of hUCMSC-ex against I/R and H/R in our study. Additionally, hUCMSC-ex contributes to improve myocardial repair after acute myocardial infarction [27]. Considering the therapeutic effects of hUCMSC-ex on cardiovascular diseases, this study further emphasized its action in I/R rat model and H/R models.

Inhibition of lncRNA UCA1 is also reported to retrain the growth and tube formation of microvascular endothelial cells; whereas, overexpression of lncRNA UCA1 is conducive to the angiogenic function of endothelial cells [28]. The H/R injury was aggravated when lncRNA UCA1 was silenced in a co-culture system of CMECs with hUCMSC-ex, as demonstrated by reduced viability, invasion and migration abilities, and enhanced apoptosis of H/R-injured CMECs. Consistently, lncRNA UCA1 facilitates the growth of human pulmonary artery smooth muscle cells and suppresses hypoxia-induced apoptosis, thus presenting as a promising target for treatment of hypoxic pulmonary hypertension [29]. Further, our data suggested that hUCMSC-ex inhibited H/R-induced autophagy in CMECs through transferring lncRNA UCA1, which is consistent with the finding reported by Gao *et al*. that lncRNA UCA1 reduces autophagy-induced cell death [30]. In addition, lncRNA UCA1 upregulated by morphine postconditioning contributes to inhibiting autophagy in I/R-induced cardiac injury [31]. Another study has also suggested that lncRNA UCA1 protected against I/R injury through suppression of I/R-triggered oxidative stress and mitochondria dysfunction [32]. Base on those findings, it is rational to conclude that hUCMSC-ex transmits the lncRNA UCA1 to maintain its function.

LncRNA UCA1 controls mRNA degradation by RNA-RNA competitive interactions [20]. This study proposed a regulatory axis implicated in the protective function of hUCMSCs-ex. In this network, lncRNA UCA1 could competitively bind to miR-143 to upregulate Bcl-2. The relationship between lncRNA UCA1 and miR-143 has been revealed in the progression of both breast cancer and bladder cancer [33, 34]. Also, the present study demonstrated that CMEC H/R injury was deteriorated with gain of function of miR-143 in H/R cell model by reducing viability but enhancing apoptosis of CMECs. Upregulation of miR-143 induces apoptosis and necrosis and aggravates the cardiac ischemia-induced mitochondrial impairment [35]. Similar with our study, inhibition of miR-143 by circular RNA DLGAP4 ameliorates I/R-induced apoptosis of cardiomyocytes *via* upregulating Bcl-2 [36]. Consistently, downregulation of miR-143 rescued cardiomyocytes from apoptosis [21]. Meanwhile, inhibition of miR-143 protects rat neurons against ischemic brain injury [37]. In a similar regulatory way, lncRNA UCA1 relieves the miR-26a-caused inhibition of PTEN to inhibit proliferation of vascular smooth muscle cells [38].

Taken together, this study elaborated a protective role of exosomal lncRNA UCA1 derived from hUCMSCs against H/R injury in CMECs through the miR-143/Bcl-2/Beclin-1 axis. The molecular mechanisms evidenced by our study contribute to better understanding of therapeutic effects of hUCMSC-ex on cardiovascular diseases. The current application is still limited by methods for efficiently isolating high yields of pure exosomes. Hence, more attention should be made for optimizing exosome isolation protocols.

## MATERIALS AND METHODS

### Ethics statement

The experiment was approved by the Ethics Committee of the Second Affiliated Hospital of Harbin Medical University and conducted in compliance with the Helsinki declaration. All individuals signed informed written consent documents. The experiments involving animals were performed with the approval of the Laboratory Animals and Care Committee (IACUC).

### Isolation and identification of hUCMSCs

Fresh human umbilical cords from parturient women after cesarean delivery in the Second Affiliated Hospital of Harbin Medical University were collected for this study. The umbilical cords were immersed in fetal bovine serum (FBS) (Thermo Fisher Scientific, Waltham, MA, USA) supplemented with 1% streptomycin and penicillin. Excess blood was removed. hUCMSCs were extracted using trypsin detachment and incubated at 37° C with 5% CO_2_. When cells reached approximately 80% confluence, hUCMSCs were passaged. Cell morphology was observed under optical microscope (Olympus, Tokyo, Japan). Flow cytometry was used to detect hUCMSCs surface markers CD29, CD44, HLA-I, CD34, CD38 and HLA-DR. The neurogenic induction of hUCMSCs was observed by immunofluorescence staining. The adipogenic differentiation was evaluated using oil red O staining. Osteogenic differentiation was assessed using alizarin red staining. Osteogenic and adipogenic induction reagents were purchased from Gibco (Grand Island, NY, USA). Neurogenic induction reagent was purchased with Shanghai Ricky Bio Technology Co., Ltd (Shanghai, China). Each induction process was carried out in strict accordance with the instructions of the kit.

### Isolation and identification of exosomes from hUCMSCs

Cells at passage 3 in the logarithmical growth phase were starved. The cell supernatant was ultra-centrifuged at 20,000 rpm at 4° C for 20 min. The collected supernatant was further filtered through a 0.22 μm sterile membrane and then ultra-centrifuged at 50000 rpm at 4° C for 1 h. After centrifugation, white pellet was observed at the bottom of the tube. The supernatant was discarded and the pellet was resuspended in PBS, followed by centrifugation at 3000 rpm at 4° C for 10 min. The collected supernatant was purified exosomes.

The particle size of exosomes was measured by Zetasizer Nano series-Nano-ZS instrument (Zetasizer Nano ZS, Hangzhou Neoline Technology Co., Ltd.) according to standard operating procedures.

The morphology of exosomes was observed under transmission electron microscope (TEM) (Olympus). Then 10 μL of the purified exosomes were added to the carrier copper grid for 1 min, and 30 μL of 20 mL/L phosphotungstic acid solution was added onto the copper grid, for negative staining for 5 min at room temperature. Afterward, the copper grid was dried under the incandescent lamp. The images were captured under the TEM.

### Culture and identification of CMECs

The left ventricle of Sprague Dawley (SD) rats was aseptically isolated, and then myocardial tissues were cut into 1 mm^3^ tissue blocks. The tissues were detached with 0.25% type II collagenase and trypsin for 10 min each, followed by centrifugation at 1000 rpm and resuspension. The suspended tissues were cultured in culture flasks. The medium was renewed after differential adhesion for 6 h. Finally, the collected cells were cultured at 37° C in a 50 mL/L CO_2_ incubator.

After 7 days of trypsinization, the cells were adjusted to 1 × 10^5^/mL and seeded in 24-well microplates. After incubation for 24 h at 37° C in a 5% CO_2_ incubator, the cells were cultured with Dil-labeled acetylated low density lipoprotein (Dil-Ac-LDL) (Invitrogen, Carlsbad, CA, USA) for 8-10 h. The uptake of Dil-ac-LDL by live cells was observed under a fluorescence microscope.

### Induction of H/R injury and cell grouping

After detachment, CMECs at passage 3 were seeded in 6-well microplates at a density of 1×10^6^/well for 24 h. Then the culture medium was replaced with saturated D-Hanks containing 950 mL/L N2 and 50 mL/L CO_2_. Next, the CMECs were incubated in the hypoxic incubator containing 940 mL/L N2, 50 mL/L CO_2_ and 10 mL/LO_2_ for 2 h at 37° C. The incubation was terminated, followed by 3 times of washes with PBS. The CMECs were re-oxygenated with Dulbecco's modified Eagle's medium (DMEM) (ie, re-oxygenation solution) (Hyclone, Logan, UT, USA) containing 20% FBS in a normal incubator (950 mL/L O_2_ and 50 mL/L CO_2_) for 4 h at 37° C to establish an H/R injury model.

CMECs cultured with only DMEM containing 20% FBS served as controls. Before hypoxic treatment, CMECs were pretreated with hUCMSCs-ex at different concentrations (40 μg/mL, 80 μg/L, 160 μg/mL) for 2 h, followed by H/R.

The isolated hUCMSCs were transfected with negative control (NC) siRNA (si-NC group) or siRNA targeting UCA1 (si-UCA1 group). The corresponding exosomes in each group were extracted using ultracentrifugation and acted on H/R injury modeled CMECs, and then named as hUCMSCs-ex/si-NC group and hUCMSCs-ex/si-UCA1 group, with hUCMSCs-ex as the control. The cells from the H/R CMEC injury model were transfected with mimic NC, inhibitor NC, miR-143 mimic or miR-143 inhibitor (all purchased from Shanghai Genechem Co., Ltd., Shanghai, China). The transfection was conducted according to the instructions of Lipofectamine^TM^ 2000 kit (Invitrogen). The expression after transfection was detected using RT-qPCR to verify the transfection efficiency.

### Cell count kit 8 (CCK-8) assay

CMECs were seeded in 96-well microplates at a density of 1 × 10^6^ per well, grouped and treated as described above. The CMECs were incubated with 10 μL of CCK-8 solution (Dojindo, Tokyo, Japan) in a cell culture incubator for 4 h. The absorbance was measured at a wavelength of 450 nm using a microplate reader.

### Transwell assay

The Transwell chambers pre-coated with Matrigel (354230, BD Biosciences, Franklin Lakes, NJ, USA) were placed in 24-well microplates. CMECs suspension was adjusted to 1 × 10^5^ cells/L. Then 200 μL cell suspensions were added to the apical Transwell chambers at 37° C for 24 h. The cells in the apical chambers were carefully wiped off with a cotton swab. The cells were stained with 1 g/L crystal violet, and observed under an inverted microscope (Leica, Solms, Germany) and 5 randomly selected fields of view were photographed. The number of CMECs in each field of view was counted and averaged.

### Scratch test

CMECs in the logarithmic growth phase were seeded into pre-marked 6-well microplates at a density of 5 × 10^8^ cells/L and dispersed evenly. The scratches were made perpendicular to the bottom surface of the microplates in each well using a 200 μL pipette. The scratches were perpendicular to the marks in the microplates. After the floating cells were washed away with PBS, the CMECs were cultured with serum-free DMEM, and the scratches were recorded at 0 h and 24 h after cell culture. The experiment was repeated 3 times.

### Terminal deoxyribonucleotidyl transferase (TdT)-mediated dUTP-digoxigenin nick end labeling (TUNEL) assay

The cell suspension was seeded into confocal culture dishes and placed in 50 mL/L CO_2_ cell incubators at 37° C for routine culture. When the cell confluence was above 80%, the TUNEL staining was performed according to the instructions of TUNEL kit (R&D system, Minneapolis, MN, USA). Finally, the nuclei were stained with 10 μg/mL 4',6-diamidino-2-phenylindole (DAPI; Wuhan Google Biotech Co., Ltd., Wuhan, China) for 10 min. The apoptotic cells were observed under a confocal microscope (Olympus) to calculate the TUNEL-positive rate.

### Flow cytometry

After CMECs were subjected to H/R treatment, the reoxygenation solution was removed and CMECs were trypsinized. Then CMECs were collected and centrifuged at 1200 rpm for 8 min. The supernatant was discarded. CMECs were stained with PI-Annexin V staining (Bestbio Biotechnology Co. Ltd, Shanghai, China) according to the kit, and apoptosis rate was detected on a flow cytometer (Partec, Munster, Germany).

### Tube formation assay

CMECs were cultured for 24-28 h at 37° C with 5% CO_2_ for angiogenesis analysis. One day before the experiment, the Matrigel from the -20° C refrigerator was melted at a relatively low temperature. Then, 50 μL Matrigel was added to each well of 96-well microplates using a pipette, and placed in the incubators at 37° C for 40 min. CMECs at passage 2 were seeded in 96-well microplates at a density of 2 × 10^4^ cells per well, and cultured at 37° C for 18 h. The tube formation ability was observed and photographed. The experiment was performed 3 times and the number of tubes was counted.

### Immunofluorescence staining

CMECs were cultured on 12-well microplates at about 1 × 10^5^ cells/slide for 3 days, following different treatments in each group. Next, cells were fixed with polyformaldehyde at room temperature for 10 min, and penetrated with 0.1% Triton X-100 at room temperature for 10 min. After being sealed at room temperature for 60 min with 10% goat serum, cells were incubated with primary antibody rabbit anti-rat LC3 (1 μg/mL, ab48394, abcam) at 4° C overnight. In the dark, cells were incubated with IgG H&L (Alexa Fluor^®^ 488) secondary antibody (1:200, ab150077, abcam) at 37° C for 60 min. The nuclei were stained with DAPI for 5 min. After that, 10 μL anti-fluorescence quenching sealing reagents were dropped on the cover glass. The cells were stored in a light-proof wet box at 4° C and photographed under a confocal laser microscope.

### A rat model of I/R injury

Male clean SD rats weighing 220-260 g provided by the Experimental Animal Center of Guangzhou University of Chinese Medicine [certificate No. SYXK (Yue) 2017-0179]. Some rats were sham-operated with only threading, but no ligation of left anterior descending (LAD) coronary artery. The LAD of other rats were ligated for 30 min, followed by 120-min reperfusion to establish the I/R injury model. The rat model of I/R injury was identified by myocardial histopathological changes using hematoxylin-eosin (HE) staining.

After model identification, the rats with I/R injury were injected with above extracted hUCMSCs-ex at different dosages (0 μg/kg, 120 μg/kg, 140 μg/kg and 160 μg/kg, 8 rats per dosage).

### TEM observation

The heart apex tissues (1 mm^3^) of rats were excised and fixed in citrate, dehydrated and embedded. Then embedded tissues were cut into slices at 70 nm, and subjected to uranyl acetate-lead citrate staining. The stained tissues were photographed under the TEM.

### Histological examination

The HE staining was conducted for histological observation of myocardial tissues. In brief, the tissues 0.5 mm below the ligation were fixed with 4% paraformaldehyde, and dehydrated with gradient alcohols. Next, paraffin-embedded tissues were sliced at 4 μm, and stained successively with hematoxylin and eosin (Beijing Solarbio Science & Technology Co., Ltd., Beijing, China).

The myocardial tissue sections were dewaxed and rehydrated, treated with 20 μg/mL proteinase K, and incubated with 50 μL prepared DNase1 working solution for 10 min at room temperature in a wet box. The tissues were stained with TUNEL reaction solution (Wuhan Servicebio Co., Ltd., Wuhan, Hubei, China), and developed with prepared diaminobenzidine (DAB), which was stopped by PBS. After hematoxylin counter-staining and 1% hydrochloric acid alcohol differentiation, the tissues turned to blue under tap water for 10 min. After dehydration and clearing, the tissues were sealed with neutral gum and TUNEL-positive rate was calculated.

### Enzyme-linked immunosorbent assay (ELISA)

After 8 weeks of exosome injection, rats were anesthetized with excessive sodium pentobarbital. A total of 15 mL blood was collected from the abdominal aorta, and allowed to stand on ice for 30 min. After the blood was centrifuged at 4500 rpm for 15 min, the supernatant was stored at low temperature. Creatine kinase (CK), high-density lipoprotein (HDL), thrombomodulin (TM), von Willebrand factor (vWF) levels in sera were determined according to the instructions of ELISA kits (Nanjing SenBeiJia Biological Technology Co., Ltd., Nanjing, Jiangsu, China).

### Circulating endothelial cell (CEC) counting

Flow cytometry was used to count the number of circulating endothelial cells. The specific operation was performed in accordance with the operating procedures. The white blood cells (WBCs) were counted using an automatic blood cell counter. The number of CECs (cells/μL) = the number of WBCs × the percentage of each quadrant.

### Reverse transcription-quantitative polymerase chain reaction (RT-qPCR)

Total RNA was extracted from cells and tissues using TRIzoL reagent (Invitrogen). After determination of RNA concentration and purity, the cDNA was synthesized using a reverse transcription kit (GeneCopoeia, Rockville, MD, USA). The expression of genes in Table 1 was quantified using a SYBR PCR Master Mix kit (Applied Biosystems, Foster City, CA, USA), and U6 or β-actin was used as an internal reference. The relative expression of target gene was expressed as 2^–ΔΔCt^. Primers used in the experiments were designed using the Primer 3Plus website and synthesized by Suzhou GENEWIZ Biotechnology Co., Ltd (Suzhou, China). The experiment was repeated 3 times.

**Table 1:** Primer sequences for RT-qPCR.

**Primer**	**Sequence**
lncRNA UCA1-F	CTCTCCATTGGGTTCACCATTC
lncRNA UCA1-R	GCGGCAGGTCTTAAGAGATGAG
miR-143-F	CCGCGCGTGAGATGAAGCACTG
miR-143-R	ATCCAGTGCAGGGTCCGAGG
U6-F	CTCGCTTCGGCAGCACA
U6-R	GTGTCGTGGAGTCGGCAA
Bcl2-F	TGAACCGGCATCTGCACAC
Bcl2-R	CGTCTTCAGAGACAGCCAGGAG
Beclin-1-F	AGGAACTCACAGCTCCATTAC
Beclin-1-R	AATGGCTCCTCTCCTGAGTT
β-actin-F	GTCATTCCAAATATGAGAGATGCGT
β-actin-R	GCTATCACCTCCCCTGTGTG

**Note:** RT-qPCR, reverse transcription-quantitative polymerase chain reaction; F, forward; R, reverse; lncRNA, long non-coding RNA; miR-143, microRNA-143; Bcl2, B-cell leukemia/lymphoma 2.

### Western blot assay

The cells were lysed using RIPA lysis buffer, and the tissues were homogenized. The proteins were extracted and quantified by bicinchoninic acid method. The proteins were transferred to a new 1.5 mL centrifuge tube. Protein electrophoresis was carried out using a polyacrylamide gel, and transferred onto a membrane at a 90 mA constant current. After being blocked with 50 g/L milk for 1 h, the membranes were incubated with primary antibodies (Table 2) overnight. Next day, the corresponding secondary antibody was added for incubation. Proteins were visualized with enhanced chemiluminescence, and photographed with Bio-Rad digital image system (Bio-Rad, Berkeley, CA, USA). In addition, exosome marker proteins CD63 and CD81 were detected by Western blot analysis.

**Table 2:** Antibodies for western blot assay.

**Name**	**No. and company**	**Dilution ratio**
CD81	ab79559, ABcam	1 μg/mL
CD63	ab179473, ABcam	1: 1000
Bcl-2	ab32124, ABcam	1: 1000
Beclin-1	ab207612, ABcam	1: 2000
Cleaved Caspase-3	ab211631, ABcam	1: 1000
Bax	ab234662, ABcam	1: 5000
LC3	ab48394, ABcam	1: 1000
MDM2	ab38618, ABcam	1:1000
P53	ab26, ABcam	2μg/mL
p-P53	ab33889, ABcam	1/1000
β-actin	ab179467, ABcam	1: 5000

**Note:** Bcl-2, B-cell leukemia/lymphoma 2; LC3 II, light chain 3; P62, hypothetical protein.

### RNA pull-down

A total of 50 μL magnetic beads, 20 mM Tris buffer (pH = 7.5), and 1× RNA Capture Buffer were mixed in an RNase-free EP tube. Then 50 pmoL biotin-labeled miR-143 was bonded to the beads, and incubated at 37° C for 2 h. Next, the beads were incubated with 2 μL DNase I at 37° C for 15 min, which was terminated with 2 μL of 0.2 M ethylenediaminetetracetic acid (EDTA) (pH = 8.0). Next, 1 μg biotin-labeled RNA was mixed with Structure Buffer (10 mM Tris, pH=7.0, 0.1 M KCl, 10 mM MgCl_2_), and the mixture was heated at 95° C for 2 min, ice-cooled for 3 min, and allowed to stand at room temperature for 30 min. Cell lysate (containing about 1 mg protein) was added to the magnetic bead-RNA mixture, and treated with RNase inhibitor at room temperature for 1 h. The supernatant was collected (as a negative control of the system) after centrifugation at low speed. The RNA was eluted with Wash Buffer II 3 times, 1 mL each time.

### Dual luciferase reporter assay

The lncRNA UCA1 and Bcl-2 3'UTR sequences containing miR-143 binding site were respectively synthesized, and the lncRNA UCA1 and Bcl-2 3'UTR wild type (WT) plasmids (UCA1-WT and Bcl-2-WT), and the lncRNA UCA1 and Bcl-2 3'UTR mutant (MUT) plasmids (UCA1-MUT and Bcl-2-MUT) were constructed. The constructed plasmids were co-transfected with mimic NC or miR-34a mimic, respectively into 293T cells (ATCC, Manassas, Virginia, USA). After 48 h of transfection, cells were harvested and lysed, and luciferase activity was measured using a luciferase assay kit (BioVision Inc., San Francisco, CA, USA) and Glomax 20/20 luminometer (Promega, Madison, WI, USA).

### Immunofluorescence and fluorescent *in situ* hybridization

Two-color fluorescence in-situ hybridization was used to further determine the relationship between miR-143 and Bcl2. The probe for detecting miR-143 was marked with red fluorescence, and the probe for detecting BCL2 was marked with green fluorescence. RNA probe and fluorescence in-situ hybridization kit were purchased from Beijing GP Medical Technology Co., Ltd (Beijing, China). The specific operations were strictly in accordance with the kit instructions.

### Statistical analysis

The data were analyzed by SPSS 21.0 statistical software (IBM Corp., Armonk, NY, USA). The Kolmogorov-Smirnov test checked whether the data were in normal distribution. The results were expressed as mean ± standard deviation. Comparisons between two groups were performed using the t test, and those among multi-groups were analyzed using one-way ANOVA or two-way ANOVA. The post-test was performed with Tukey's multiple comparisons test. A two-tailed *p* value less than 0.05 indicates statistically significant.

## References

[1] De Pascali F, Hemann C, Samons K, Chen CA, Zweier JL. Hypoxia and reoxygenation induce endothelial nitric oxide synthase uncoupling in endothelial cells through tetrahydrobiopterin depletion and S-glutathionylation. Biochemistry. 2014; 53:3679–88. 10.1021/bi500076r 24758136 PMC4053070

[2] Coimbra-Costa D, Alva N, Duran M, Carbonell T, Rama R. Oxidative stress and apoptosis after acute respiratory hypoxia and reoxygenation in rat brain. Redox Biol. 2017; 12:216–25. 10.1016/j.redox.2017.02.014 28259102 PMC5334548

[3] Turer AT, Hill JA. Pathogenesis of myocardial ischemia-reperfusion injury and rationale for therapy. Am J Cardiol. 2010; 106:360–68. 10.1016/j.amjcard.2010.03.032 20643246 PMC2957093

[4] Hausenloy DJ, Yellon DM. Myocardial ischemia-reperfusion injury: a neglected therapeutic target. J Clin Invest. 2013; 123:92–100. 10.1172/JCI62874 23281415 PMC3533275

[5] Loyer X, Vion AC, Tedgui A, Boulanger CM. Microvesicles as cell-cell messengers in cardiovascular diseases. Circ Res. 2014; 114:345–53. 10.1161/CIRCRESAHA.113.300858 24436430

[6] Vicencio JM, Yellon DM, Sivaraman V, Das D, Boi-Doku C, Arjun S, Zheng Y, Riquelme JA, Kearney J, Sharma V, Multhoff G, Hall AR, Davidson SM. Plasma exosomes protect the myocardium from ischemia-reperfusion injury. J Am Coll Cardiol. 2015; 65:1525–36. 10.1016/j.jacc.2015.02.026 25881934

[7] Li JW, Wei L, Han Z, Chen Z. Mesenchymal stromal cells-derived exosomes alleviate ischemia/reperfusion injury in mouse lung by transporting anti-apoptotic miR-21-5p. Eur J Pharmacol. 2019; 852:68–76. 10.1016/j.ejphar.2019.01.022 30682335

[8] Dorronsoro A, Robbins PD. Regenerating the injured kidney with human umbilical cord mesenchymal stem cell-derived exosomes. Stem Cell Res Ther. 2013; 4:39. 10.1186/scrt187 23680102 PMC3706756

[9] Lai RC, Yeo RW, Lim SK. Mesenchymal stem cell exosomes. Semin Cell Dev Biol. 2015; 40:82–88. 10.1016/j.semcdb.2015.03.001 25765629

[10] D’Souza-Schorey C, Schorey JS. Regulation and mechanisms of extracellular vesicle biogenesis and secretion. Essays Biochem. 2018; 62:125–33. 10.1042/EBC20170078 29666210

[11] Haemmig S, Feinberg MW. Targeting LncRNAs in cardiovascular disease: options and expeditions. Circ Res. 2017; 120:620–23. 10.1161/CIRCRESAHA.116.310152 28209793 PMC5325063

[12] Simion V, Haemmig S, Feinberg MW. LncRNAs in vascular biology and disease. Vascul Pharmacol. 2019; 114:145–56. 10.1016/j.vph.2018.01.003 29425892 PMC6078824

[13] Cui C, Li Z, Wu D. The long non-coding RNA H19 induces hypoxia/reoxygenation injury by up-regulating autophagy in the hepatoma carcinoma cells. Biol Res. 2019; 52:32. 10.1186/s40659-019-0239-2 31196153 PMC6567522

[14] Treiber T, Treiber N, Meister G. Regulation of microRNA biogenesis and its crosstalk with other cellular pathways. Nat Rev Mol Cell Biol. 2019; 20:5–20. 10.1038/s41580-018-0059-1 30728477

[15] Wang QS, Zhou J, Li X. LncRNA UCA1 protects cardiomyocytes against hypoxia/reoxygenation induced apoptosis through inhibiting miR-143/MDM2/p53 axis. Genomics. 2020; 112:574–80. 10.1016/j.ygeno.2019.04.009 30998966

[16] Zhang B, Wu X, Zhang X, Sun Y, Yan Y, Shi H, Zhu Y, Wu L, Pan Z, Zhu W, Qian H, Xu W. Human umbilical cord mesenchymal stem cell exosomes enhance angiogenesis through the Wnt4/β-catenin pathway. Stem Cells Transl Med. 2015; 4:513–22. 10.5966/sctm.2014-0267 25824139 PMC4414225

[17] Huang KY, Wang JN, Zhou YY, Wu SZ, Tao LY, Peng YP, Que JQ, Xue YJ, Ji KT. Antithrombin III alleviates myocardial ischemia/reperfusion injury by inhibiting excessive autophagy in a phosphoinositide 3-Kinase/Akt-dependent manner. Front Pharmacol. 2019; 10:516. 10.3389/fphar.2019.00516 31133861 PMC6522837

[18] Lim W, Kim HS. Exosomes as therapeutic vehicles for cancer. Tissue Eng Regen Med. 2019; 16:213–23. 10.1007/s13770-019-00190-2 31205851 PMC6542887

[19] Zhang Z, Li H, Cui Z, Zhou Z, Chen S, Ma J, Hou L, Pan X, Li Q. Long non-coding RNA UCA1 relieves cardiomyocytes H9c2 injury aroused by oxygen-glucose deprivation via declining miR-122. Artif Cells Nanomed Biotechnol. 2019; 47:3492–99. 10.1080/21691401.2019.1652630 31432699

[20] Barbagallo C, Brex D, Caponnetto A, Cirnigliaro M, Scalia M, Magnano A, Caltabiano R, Barbagallo D, Biondi A, Cappellani A, Basile F, Di Pietro C, Purrello M, Ragusa M. LncRNA UCA1, upregulated in CRC biopsies and downregulated in serum exosomes, controls mRNA expression by RNA-RNA interactions. Mol Ther Nucleic Acids. 2018; 12:229–41. 10.1016/j.omtn.2018.05.009 30195762 PMC6023947

[21] Tian S, Zhao W, Yang D, Yu Y, Zou J, Liu Z, Du Z. Atorvastatin inhibits miR-143 expression: a protective mechanism against oxidative stress in cardiomyocytes. Int J Cardiol. 2016; 211:115–18. 10.1016/j.ijcard.2016.02.141 26995052

[22] Zhang H, Zhong Z, Chen W, Bai H, Xiao Y, Gu Y, Lu S. [Effects of electroacupuncture at different time during reperfusion on the expression of Bcl-2 and Beclin1 in myocardial tissue in rats with myocardial ischemia reperfusion injury]. Zhongguo Zhen Jiu. 2018; 38:1195–200. 10.13703/j.0255-2930.2018.11.015 30672201

[23] Zhang X, Zhang Q, Li W, Nie D, Chen W, Xu C, Yi X, Shi J, Tian M, Qin J, Jin G, Tu W. Therapeutic effect of human umbilical cord mesenchymal stem cells on neonatal rat hypoxic-ischemic encephalopathy. J Neurosci Res. 2014; 92:35–45. 10.1002/jnr.23304 24265136

[24] Zhu J, Liu Q, Jiang Y, Wu L, Xu G, Liu X. Enhanced angiogenesis promoted by human umbilical mesenchymal stem cell transplantation in stroked mouse is Notch1 signaling associated. Neuroscience. 2015; 290:288–99. 10.1016/j.neuroscience.2015.01.038 25637797

[25] Huang Z, Ye B, Dai Z, Wu X, Lu Z, Shan P, Huang W. Curcumin inhibits autophagy and apoptosis in hypoxia/reoxygenation-induced myocytes. Mol Med Rep. 2015; 11:4678–84. 10.3892/mmr.2015.3322 25673156

[26] Zhao Y, Sun X, Cao W, Ma J, Sun L, Qian H, Zhu W, Xu W. Exosomes Derived from Human Umbilical Cord Mesenchymal Stem Cells Relieve Acute Myocardial Ischemic Injury. Stem Cells Int. 2015; 2015:761643. 10.1155/2015/761643 26106430 PMC4461782

[27] Wang XL, Zhao YY, Sun L, Shi Y, Li ZQ, Zhao XD, Xu CG, Ji HG, Wang M, Xu WR, Zhu W. Exosomes derived from human umbilical cord mesenchymal stem cells improve myocardial repair via upregulation of Smad7. Int J Mol Med. 2018; 41:3063–72. 10.3892/ijmm.2018.3496 29484378

[28] Yin D, Fu C, Sun D. Silence of lncRNA UCA1 represses the growth and tube formation of human microvascular endothelial cells through miR-195. Cell Physiol Biochem. 2018; 49:1499–511. 10.1159/000493454 30205377

[29] Zhu TT, Sun RL, Yin YL, Quan JP, Song P, Xu J, Zhang MX, Li P. Long noncoding RNA UCA1 promotes the proliferation of hypoxic human pulmonary artery smooth muscle cells. Pflugers Arch. 2019; 471:347–55. 10.1007/s00424-018-2219-8 30353369

[30] Gao M, Li C, Xu M, Liu Y, Liu S. LncRNA UCA1 attenuates autophagy-dependent cell death through blocking autophagic flux under arsenic stress. Toxicol Lett. 2018; 284:195–204. 10.1016/j.toxlet.2017.12.009 29248574

[31] Chen Z, Liu R, Niu Q, Wang H, Yang Z, Bao Y. Morphine postconditioning alleviates autophage in ischemia-reperfusion induced cardiac injury through up-regulating lncRNA UCA1. Biomed Pharmacother. 2018; 108:1357–64. 10.1016/j.biopha.2018.09.119 30372838

[32] Chen J, Hu Q, Zhang BF, Liu XP, Yang S, Jiang H. Long noncoding RNA UCA1 inhibits ischaemia/reperfusion injury induced cardiomyocytes apoptosis via suppression of endoplasmic reticulum stress. Genes Genomics. 2019; 41:803–10. 10.1007/s13258-019-00806-w 30877641

[33] Luo J, Chen J, Li H, Yang Y, Yun H, Yang S, Mao X. LncRNA UCA1 promotes the invasion and EMT of bladder cancer cells by regulating the miR-143/HMGB1 pathway. Oncol Lett. 2017; 14:5556–62. 10.3892/ol.2017.6886 29113184 PMC5662909

[34] Tuo YL, Li XM, Luo J. Long noncoding RNA UCA1 modulates breast cancer cell growth and apoptosis through decreasing tumor suppressive miR-143. Eur Rev Med Pharmacol Sci. 2015; 19:3403–11. 26439035

[35] Hong H, Tao T, Chen S, Liang C, Qiu Y, Zhou Y, Zhang R. MicroRNA-143 promotes cardiac ischemia-mediated mitochondrial impairment by the inhibition of protein kinase cepsilon. Basic Res Cardiol. 2017; 112:60. 10.1007/s00395-017-0649-7 28887629

[36] Wang S, Chen J, Yu W, Deng F. Circular RNA DLGAP4 ameliorates cardiomyocyte apoptosis through regulating BCL2 via targeting miR-143 in myocardial ischemia-reperfusion injury. Int J Cardiol. 2019; 279:147. 10.1016/j.ijcard.2018.09.023 30213603

[37] Zeng X, Liu N, Zhang J, Wang L, Zhang Z, Zhu J, Li Q, Wang Y. Inhibition of miR-143 during ischemia cerebral injury protects neurones through recovery of the hexokinase 2-mediated glucose uptake. Biosci Rep. 2017; 37:BSR20170216. 10.1042/BSR20170216 28522551 PMC6434090

[38] Tian S, Yuan Y, Li Z, Gao M, Lu Y, Gao H. LncRNA UCA1 sponges miR-26a to regulate the migration and proliferation of vascular smooth muscle cells. Gene. 2018; 673:159–66. 10.1016/j.gene.2018.06.031 29908280

